# Supplementary material for: Asymmetric Synthesis of Quaternary Hydantoins via a Palladium-Catalyzed Aza-Heck Cyclization
Source: J Am Chem Soc. 2025 Nov 14;147(49):44692–8. doi: 10.1021/jacs.5c16022 (PMC12703750; doi:10.1021/jacs.5c16022)
Supplement: Supplementary file 2 [file ja5c16022_si_002.zip › All NMR FID Files/S29/S29_AllNMR/Carbon APT/pdata/1/email_TDI01049-retake_2_1.pdf]

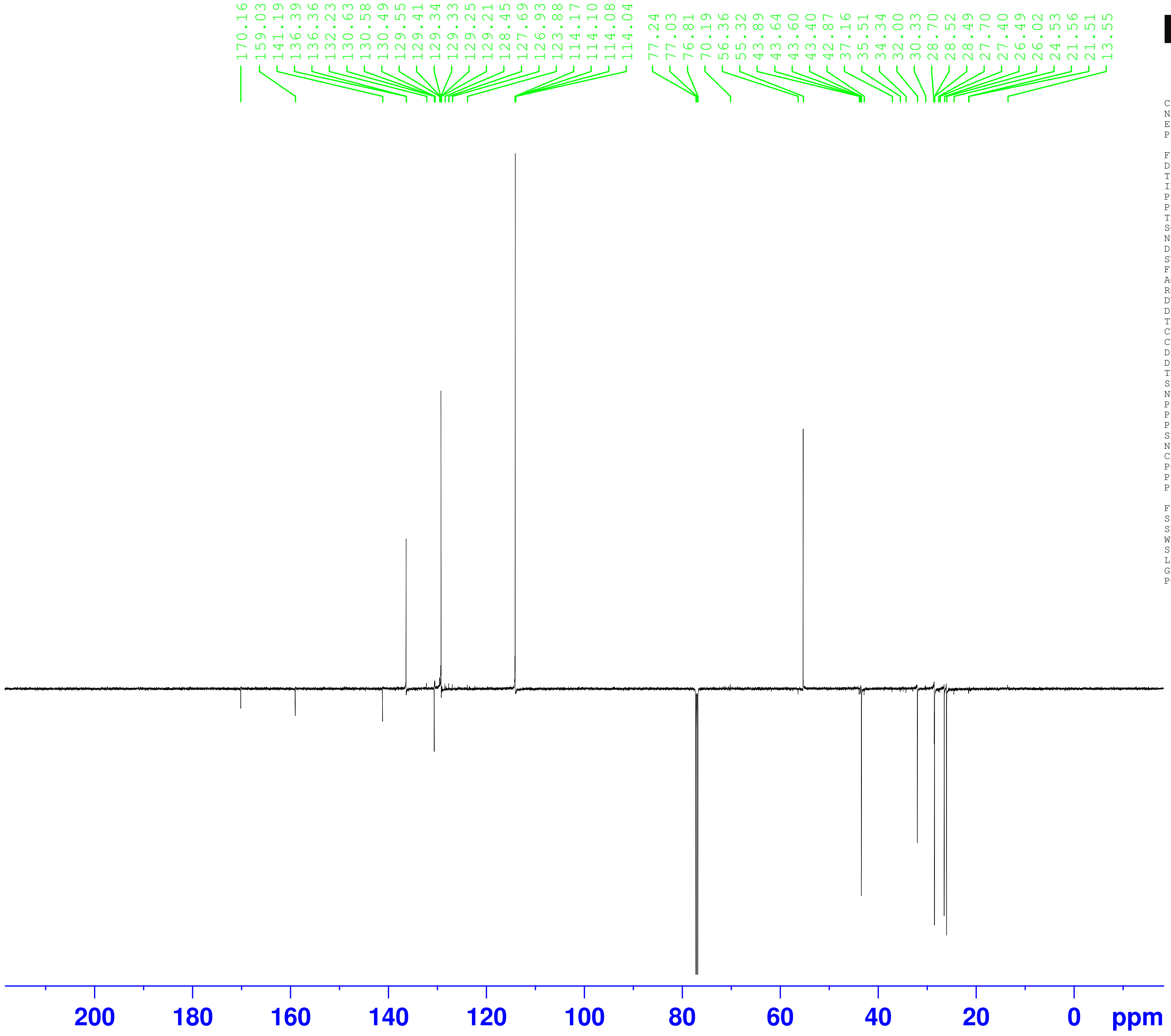


```
Current Data Parameters
NAME      TDI01049-retake
EXPNO                   2
PROCNO                  1

F2 - Acquisition Parameters
Date_            20230830
Time                23.23 h
INSTRUM   Avance Neo 600 at University of Delaware
PROBHD    Z185170_0001 (
PULPROG              jmod
TD                  65536
SOLVENT             CDCl3
NS                   1024
DS                      4
SWH             35714.286 Hz
FIDRES           1.089913 Hz
AQ              0.9175040 sec
RG                    101
DW                 14.000 usec
DE                  19.25 usec
TE                  298.0 K
CNST2         145.0000000
CNST11          1.0000000
D1             2.00000000 sec
D20            0.00689655 sec
TD0                     1
SFO1          150.9178993 MHz
NUC1                  13C
P1                  11.78 usec
P2                  23.56 usec
PLW1         106.00000000 W
SFO2          600.1324005 MHz
NUC2                   1H
CPDPRG[2          waltz65
PCPD2               70.00 usec
PLW2           6.90000010 W
PLW12          0.09034800 W

F2 - Processing parameters
SI                  32768
SF            150.9028090 MHz
WDW                    EM
SSB                     0
LB                   1.00 Hz
GB                      0
PC                   1.40
```
